# Evaluation of Wound Healing Potential of *Bauhinia purpurea* Leaf Extracts in Rats

**DOI:** 10.4103/0250-474X.62250

**Published:** 2010

**Authors:** K. V. Ananth, M. Asad, N. Prem Kumar, S. M. B. Asdaq, G. S. Rao

**Affiliations:** Department of Pharmacology, Krupanidhi College of Pharmacy, Chikkabellandur, Carmelaram post, Varthur Hobli, Bangalore-560 035, India

**Keywords:** *Bauhinia purpurea*, burn wound, dead space, excision wound, incision wound, wound healing

## Abstract

The present study was carried out to evaluate the effect of methanol and chloroform extracts of *Bauhinia purpurea* on experimentally induced excision, incision, burn and dead space wound models in Sprague Dawley rats. Formulations of methanol and chloroform extracts of *Bauhinia purpurea* were prepared in carbopol and simple ointment base at concentrations of 2.5% and 5% and applied to the wounds. In the excision and burn wound models, animals treated with high doses of methanol and chloroform showed significant reduction in time taken for epithelization and wound contraction (50%) compared to control. A significant increase in breaking strength was found in incision wound model with methanol and chloroform extracts compared to their respective bases. In the dead space wound model, methanol and chloroform extract treatment (100 and 500 mg/kg) orally produced a significant increase in the breaking strength, dry tissue weight and hydroxyproline content of the granulation tissue when compared to control. Among the extracts, methanol extract exhibited more activity followed by the chloroform extract. In conclusion, the present study indicated that *Bauhinia purpurea* leaves exhibited wound healing activity.

Subsequent to skin and other soft tissue injuries, healing of wound occurs as an aspect of repair. The healing process involves inflammatory response followed by enhanced collagen synthesis beneath the dermis and epithelization. Proper management of wound with dressing, administration of pain killers, antiinflammatory drugs and promoters of wound healing augments the healing of wounds. Healing of wound includes number of stages like clotting, inflammation, granulation, fibrosis, arrangement of collagen with spasm of wound and epithelization[1].

*Bauhinia purpurea* Linn. is a flowering plant belonging to the family Caesalpiniaceae, native to South China, Malaysia and India. The plant is popularly known as *khairwal* in India, where the leaves of the plant are used mainly as plates for serving meal. The bark was reported as antimycobacterial, antimalarial, antifungal, cytotoxic, and antiinflammatory activities[2]. The leaves were reported to possess antinociceptive, antiinflammatory and antipyretic properties[3], while the stem was found to have anti-diabetic and adrenergic properties[4]. Bauhiniastatins, isolated from leaves and bark was reported to inhibit human cancer cell lines[5]. Even though, traditionally, leaves of *Bauhinia purpurea* were extensively used for the treatment of variety of wounds[6], no scientific data in its support is available. The present study was designed to evaluate wound healing activity of chloroform (CEBP) and methanol (MEBP) extracts of *Bauhinia purpurea* on experimentally induced wounds in rats.

The leaves of *Bauhinia purpurea* were collected from the rural areas of Bangalore, Karnataka, and were authenticated at the Regional Research Institute, Bangalore (RRCBI-AP3273). The leaves were air dried and powdered. The powdered leaves were extracted using methanol and chloroform as solvents to get MEBP and CEBP, respectively in a Soxhlet apparatus until complete extraction. The two different extracts were collected and vacuum dried followed by desiccation to get the constant weight. Both MEBP and CEBP were subjected to qualitative analysis for the various phytoconstituents such as alkaloids, carbohydrates, glycosides, phytosterols, saponins, tannins, proteins, amino acids and flavonoids. The experimental protocols were approved by Institutional Animal Ethics Committee (KCP/IAEC-01/2007) for the use of Sprague Dawley rats (200-250 g). The animals were maintained in an animal house recognized by the Committee for the Purpose of Control and Supervision on Experiments on Animals. Xylazine was procured from Indian Immunological Ltd. (Guntur, India). Ketamine injection was purchased from Prem Pharmaceuticals Pvt. Ltd. (Indore, India). Hydroxyproline, paradimethylamino benzaldehyde, methanol, cetostearyl alcohol, white soft paraffin, hard paraffin and wool fat were taken from S. D. Fine Chemicals Pvt. Ltd, Mumbai, India. Sodium hydroxide (NaOH), hydrogen peroxide (H_2_O_2_) and copper sulphate (CuSO_4_) were obtained from Nice Chemicals Pvt. Ltd. Mumbai, India. Carboxymethylcellulose (CMC) and Carbopol 940 was purchased from Loba Chemie Mumbai, India. Choloform was obtained from Merck Specialities Pvt. Ltd. (Mumbai, India). Tween 80 was purchased from Thomas Baker, Mumbai. Hydrochloric acid (HCl) was bought from Ranbaxy Fine Chemicals Pvt. Ltd Mumbai, India. The oral dose of MEBP and CEBP for dead space wound was selected based on oral acute toxicity test. The oral acute toxicity test was carried out as per OPPTS guidelines (Office of Prevention, Pesticide and Toxic Substance)[7]. The overnight fasted mice were given 2 g/kg and 5 g/kg orally. There was no mortality with both doses of methanol and chloroform extracts. Therefore, 1/10^th^ and 1/50^th^ of 5 g/kg corresponding to 500 and 100 mg/kg were chosen as high and low doses respectively. The suspensions of extract were made in distilled water just before treatment using Tween 80 (2%) and acacia (5%) as suspending/emulsifying agents for CEBP and MEBP respectively. *Aloe vera* extract was used as standard drug as suspension (acacia 5%) at 300 mg/kg orally[8].

The knowledge of traditional healers was used for selecting the doses for local application in excision, incision and burn wound models. The chloroform extract was formulated as 2.5% (w/w) and 5% (w/w) in hydrophilic base namely carbopol (1%) containing methyl paraben (0.01%) and propyl paraben (0.1%)[9]. The methanol extract was formulated as 2.5% (w/w) and 5% (w/w) in hydrophobic base viz., simple ointment[10]. *Bauhinia purpurea* (BP) 2.5% (w/w) was used as a low dose and BP 5% (w/w) was used as a high dose for topical application. Since, modern medicine uses mainly antibacterial agents for treatment of wound, an herbal drug, *Aloe vera* 5%(w/w) was used as standard for topical application in excision, incision and burn wound models[11]. The methanol extract of aloe contains all the chemical constituents responsible for wound healing activity.

In excision wound model[12,13], animals were anesthetized with ketamine (100 mg/kg, i.m.) and xylazine (16 mg/kg, i.m.). Around 5 cm away from the ear and 1 cm away from vertebral column, an impression was made in anaesthetized rat. The skin from the shaved area was excised to obtain a area of about 500 square millimeter. The animals were then grouped as follows, group I- simple ointment, group II- MEBP (2.5%), group III- MEBP (5%), group IV- *Aloe vera* (5%) formulation, group V- Carbopol (1%) gel, group VI- CEBP (2.5%) and group VII- CEBP (5%). The measurement of wound area was carried out by tracing on the mm scale graph paper on 0, 2, 4, 6, 8, 10, 12, 14, 16, 18, 20 and 22 days post-wounding for determination of wound contraction-50%. Falling of scar without raw wound was considered as end point of epithelization and the days needed for this was taken as period of epithelization.

A paravertebral straight incision of 6 cm length was made on both sides of the vertebral column in the incision wound model[14–16]. The wound was closed by suturing at equidistance about 1 cm apart. Animals were treated daily from 0 to 9^th^ post-wounding day. The breaking strength of wound was measured on 10^th^ day by continuous, constant water flow technique.

Under ketamine (100 mg/kg, im) and xylazine (16 mg/kg, im) anesthesia, overnight-fasted animals were subjected to partial thickness burn wounds[17,18] by pouring hot molten wax (2 g) at 80°. Through a cylinder of 300 mm^2^ circular opening, wax was added and left on the shaven back of the animal till it gets solidified. Immediately after the injury and on subsequent days, the drugs or base was applied topically as mentioned above.

Dead space wound[19] was created by implanting subcutaneously a 2.5×5 cm polypropylene tube on either side of the lumbar region on the ventral surface of each rat. Animals received drug from 0 day to 9^th^ post wounding day. Group I- control group, group II- *Aloe vera* extract (300 mg/kg p.o.), group III- MEBP (100 mg/kg p.o.), group IV- MEBP (500 mg/kg p.o.), group V- CEBP (100 mg/kg p.o.) and group VI- CEBP (500 mg/kg p.o.). On the 10^th^ post wounding day, granulation tissue harvested on the implanted tube, was carefully dissected out along with the tube. The granulated tube was cut to get a sheet of granulation tissue, dried at 60° for 24 h, and weight was documented. To the dried tissues 5 ml of 6 N HCl was added and hydrolysates were heated at 110° for 24 h. After 24 h, the hydrolysates were neutralized using 10 N NaOH. The neutralized acid hydrolysate of the dry tissue was used for the determination of hydroxyproline. Hydroxyproline present in the acid hydrolysate of granulation tissue was oxidized by 10N sodium peroxide in the presence of copper sulfate, and when complexed with para-dimethylaminobezaldehyde, develops a pink color that was measured at 540 nm using colorimeter.

Skin irritation study[20] was carried out in rabbits. The formulation base and formulations containing two concentrations of both extracts were applied on three shaved portions of the dorsal side (about 6 cm length). After 4 h, the skin was observed for signs of inflammation and scored as follows: No erythema, no edema-0; very slight erythema, very slight edema -1; well defined erythema with slight edema -2; moderate to severe erythema with moderate edema -3 and severe erythema with severe edema -4. The study was carried on two different animals for each formulations and average of the two scores was taken as an index of skin irritation. Results were presented as mean±SEM. All groups were compared employing one-way Analysis of Variance (ANOVA) followed by Bonferroni's test[12]. The results were termed significant statistically when probability was less than 0.05 (*P*<0.05).

The present study was designed to investigate the influence of the methanol (MEBP) and chloroform (CEBP) extracts of *Bauhinia purpurea* leaf on experimentally induced wound in rats. The result demonstrates and confirms the efficacy of *Bauhinia purpurea* and thereby proves the folklore use of the herb for variety of wounds. The extracts were found to be effective at mainly three main phases of wound healing, i.e. collagenation, wound contraction and epithelization. The extract applied locally or administered orally promoted the breaking strength, wound contraction, period of epithelization and hydroxyproline concentration in different models of experimental wounds.

Four different models were used in our study to assess the wound healing effect of MEBP and CEBP extracts on various phases of wound healing. *Aloe vera* was used as a standard reference to assess the healing potency of the two extracts against their respective bases. High doses of the both extracts of *Bauhinia purpurea* leaves were found more effective when compared to *Aloe vera*.

Both high as well as low doses of MEBP and CEBP produced a significant reduction in period of epithelization and wound contraction (50%) as compared to their respective bases. Treatment with *Aloe vera* also produced significant reduction in the period of epithelization (*P*<0.001). Comparative analysis revealed that MEBP (2.5%), MEBP (5%), CEBP (5%) and *Aloe vera* had almost equal wound healing activity (Table 1). In the incision model, all treatments produced a significant increase in breaking strength when compared to their respective bases (Table 1). Topical application of both doses of MEBP and *Aloe vera* showed significant (*P*<0.05) reduction in period of epithelization and wound contraction -50% (days) compared to simple ointment base. The high dose of CEBP was found to shorten the period of epithelization (*P*<0.05) and wound contraction -50% (*P*<0.05) significantly compared to carbopol 1%, whereas, low dose of CEBP did not show similar results (Table 2) The breaking strength of 10 days old granulation tissue was significantly (*P*<0.05) promoted by both low and high doses of MEBP and CEBP as well as *Aloe vera* extract compared to control. Further, the dry tissue weight was significantly (*P*<0.05) increased by all the treatments when compared with control. The hydroxyproline content was significantly (*P*<0.05) enhanced with *Aloe vera* extract as well as high doses of both CEBP and MEBP compared to control. Moreover, the low dose of MEBP was shown to rise hydroxyproline content significantly (*P*<0.05) compared to control, while low dose of CEBP could not able to show any significant change in this parameter (Table 3). Low dose of *Bauhinia purpure* a methanol extract formulations did not showed any type of irritation, whereas, high dose produces noticeable inflammation with slight redness. On the other hand, both the low and high doses of *Bauhinia purpurea* chloroform extract formulations were not shown to elicit any type of irritation or inflammation.

**TABLE 1:** EFFECT ON PERIOD OF EPITHELIZATION AND WOUND CONTRACTION IN EXCISION AND BREAKING STRENGTH IN INCISION WOUND MODEL

Groups	Treatment	Excision wound		Incision wound Breaking strength (ml)
		
		Epithelization period (days)	Wound Contraction-50% (days)	
Group I	Simple ointment	18.66±0.42	8.033±0.30	387±9.09
Group II	MEBP (2.5%)	15.33±0.42* (−18%)	5.6±0.21* (−30.28%)	425.666±9.00* (9.99%)
Group III	MEBP (5%)	14.33±0.38* (−23.20%)	5.333±0.32* (−33.64%)	458.5±15.22* (18.475%)
Group IV	*Aloe vera* (5%)	14.66±0.42* (−21.4%)	5.566±0.15* (−30.71%)	494.333±8.81* (27.73%)
Group V	Carbopol	17.00±0.44	7.333±0.30	362.85±19.08
Group VI	CEBP (2.5%)	16.33±0.33 (−3.94%)	6.125±0.67 (−16.47%)	412±13.21 (13.81%)
Group VII	CEBP (5%)	15±0.44+ (−11.76 %)	5.8±0.22 (−20.90 %)	461.5±12.15+ (27.18%)

All values are mean±SEM, n=6

* *P*<0.05 MEBP Vs simple ointment

+ *P*<0.05, CEBP Vs carbopol; Values in parenthesis indicate % change compared to respective base; MEBP-Methanol extract of *Bauhinia purpurea* leaves; CEBP- Chloroform extract of *Bauhinia purpurea* leaves.

**TABLE 2:** EFFECT ON PERIOD OF EPITHELIZATION AND WOUND CONTRACTION IN BURN WOUND MODEL

Groups	Treatment	Burn wound	
		
		Epithelization period (days)	Wound Contraction-50% (days)
Group I	Simple ointment	15.83±0.30	6.23±0.27
Group II	MEBP (2.5%)	14.33±0.49 (−9.48%)	5.86±0.22 (−5.88%)
Group III	MEBP (5%)	12.16±0.40* (−23.14%)	4.200.12* (−32.61%)
Group IV	*Aloe vera* (5%)	13.13±0.40* (−17.25%)	3.86±0.17* (−37.98%)
Group V	Carbopol	16.500.50	6.83±0.18
Group VI	CEBP (2.5%)	16.33±0.49 (−1.03%)	6.78±0.42 (−0.74%)
Group VII	CEBP (5%)	14.500.42+ (−12.12%)	4.96±0.33+ (−27.32%)

All values are mean±SEM, n=6

* *P*<0.05 MEBP Vs simple ointment

+ *P*<0.05 CEBP Vs carbopol; Values in parenthesis indicate % change compared to respective base; MEBP-Methanol extract of *Bauhinia purpurea* leaves; CEBP- Chloroform extract of *Bauhinia purpurea* leaves.

**TABLE 3:** EFFECT ON BREAKING STRENGTH, DRY TISSUE WEIGHT AND HYDROXYPROLINE CONTENT IN DEAD SPACE WOUND

Groups	Breaking strength (g)	Dry tissue weight (g)	Concentration of hydroxyproline (μg/g of tissue)
Control	308.66±17.66	77.16±4.22	3011.83±822.54
Standard *Aloe vera* extract (300 mg/kg, p.o)	564.33±17.12* (82.82%)	206.7012.53* (167.86%)	6082.45±363.81* (101.948 %)
MEBP (100 mg/kg, p.o)	479.5010.71* (55%)	183.03±6.74* (137.19%)	4505.98±270.36* (49.6%)
MEBP (500 mg/kg, p.o)	582.66± 3.51* (75.81%)	219.41±7.82* (172.67%)	6405.76±265.54* (112.68%)
CEBP (100 mg/kg, p.o)	418.16±14.36* (35%)	139.45±5.67* (80.71%)	4252.47±130.12 (41.18%)
CEBP (500 mg/kg, p.o)	527.16±15.33* (71%)	197.22±9.40* (155.58%)	5720.91± 267.6* (89.94%)

All values are mean±SEM, n=6

* *P*<0.05 Vs control. Values in parenthesis indicate % change compared to control.

Restoration of cellular structure and tissue makes healing of wound a complex and dynamic process. Contraction causes shrinkage of wound commencing in the fibroblastic stage. The inflammatory, proliferative and maturational phases of healing is depends on the type and extent of damage, the condition of the patient's health and the potential of the tissue to repair[21]. The intervention into any one of these phases by drugs could eventually lead to either promotion or depression of the healing process[10]. Both CEBP and MEBP were effective in acceleration the healing process at higher concentrations as evident by remarkable reduction in days to complete epithelization process to bring back normal conditions in excision and burn wound models.

Enhancement of epithelial cell proliferation and collagen cell formation are the functions of growth hormone[22,23]. Growth hormone is also important for promoting the proliferation of fibroblasts[24]. In the dead space wound model, *Bauhinia purpurea* treatment increased granuloma tissue weight and breaking strength. It could be speculated that both CEBP and MEBP might act by increasing the proliferation of epithelial cell under the influence of growth hormones. The increased granuloma tissue weight and breaking strength of granulation tissue could be due to augmented synthesis of collagen under the influence of CEBP and MEBP.

Lipid peroxidation is common step in several types of injuries like burn, inflicted wound and skin ulcers. Any drug that inhibits lipid peroxidation is believed to increase the viability of collagen fibrils, increasing the strength of collagen fibers by an increase in circulation, thereby preventing the cell damage and promoting the DNA synthesis. It is reported that antioxidants, such as vitamin C and vitamin E have been shown to promote wound contraction and epithelization[25]. The free radical scavenging property of the *Bauhinia purpurea* leaves, conferred upon it by the presence of high amounts of tannin, may be responsible to the pro healing action of the extract in wound models.

The skin irritation study on the rabbit skin proved that drug in high (MEBP 5%) concentration of methanol extract produce a slight redness to the skin while it does not show any severe type of irritation when applied at low (MEBP 2.5%) concentration to the skin. Both high and low doses (CEBP 2.5% and CEBP 5%) do not show any type of irritation and inflammation.

To conclude, methanol and chloroform extracts of *Bauhinia purpurea* leaves possess good wound healing activity when applied locally or administered orally. The high doses of both extracts are more effective when applied locally and high dose of methanol extract possessed much better effect in dead space wound when administered orally. Both the extracts did not produce any substantial type of skin irritation. *Bauhinia purpurea* is having almost equal activity with *Aloe vera* in excision, incision, burn and dead space wound. However, further studies should be carried out with isolated constituent of the extract to exactly determine the lead molecule responsible for the wound healing property.
